# Safety Evaluation of *Weissella paramesenteroides* MW‐142 Isolated From Traditional Fermented Mulberry Wine

**DOI:** 10.1002/mbo3.70302

**Published:** 2026-05-11

**Authors:** Juan Huang, Yan‐yan Huang, Dong‐mei Liu, Qing‐bo Yao, Jun Tang, Su‐ping Zeng, Liu‐jun Liu

**Affiliations:** ^1^ School of Food & Pharmaceutical Science and Technology Guangzhou College of Technology and Business Guangzhou Guangdong P. R. China; ^2^ School of Food Science and Engineering South China University of Technology Guangzhou Guangdong P. R. China; ^3^ College of Food Science and Engineering Foshan University Foshan Guangdong P. R. China; ^4^ Guangdong Provincial Key Laboratory of Intelligent Food Manufacturing Foshan University Foshan Guangdong P. R. China

**Keywords:** genomic analysis, harmful metabolites, lactic acid bacteria, probiotic safety, *Weissella paramesenteroides*

## Abstract

*Weissella paramesenteroides* MW‐142 is a lactic acid bacterium (LAB) isolated from traditional fermented mulberry wine, with prior optimization of its high‐density cultivation indicating industrial potential. To ensure its safe application in food and pharmaceuticals, this study comprehensively evaluated the biosafety of MW‐142 through phenotypic and genomic analyses. An acute oral toxicity test in mice (20.0 mL/kg body weight, corresponding to 9.5 × 10^8^ CFU/mL) showed no signs of poisoning or mortality over 14 days, with histopathological analysis confirming normal organ morphology. Hemolysis tests revealed no hemolytic zones (γ‐hemolysis). Biochemical assays for harmful metabolites (indole, nitroreductase, amino acid decarboxylase, and azoreductase) were negative. Genomic screening using the Virulence Factor Database (VFDB) and the Comprehensive Antibiotic Resistance Database (CARD) identified 83 putative virulence‐associated genes; however, over half shared < 50% sequence similarity with known determinants, and no classical toxin genes (e.g., hemolysins, enterotoxins) were found. Only intrinsic, low‐risk antibiotic resistance genes were identified, and these were not associated with mobile genetic elements. These findings establish MW‐142 as a safe candidate for industrial applications, providing a scientific basis for its further development as a probiotic or biopreservative.

## Graphical abstract

Integrated safety evaluation of *Weissella paramesenteroides* MW‐142 from traditional fermented mulberry wine demonstrates no acute oral toxicity, non‐hemolytic activity, absence of harmful metabolites, and only intrinsic, non‐mobile antibiotic resistance genes, supporting its potential as a safe candidate for probiotic or biopreservative applications.

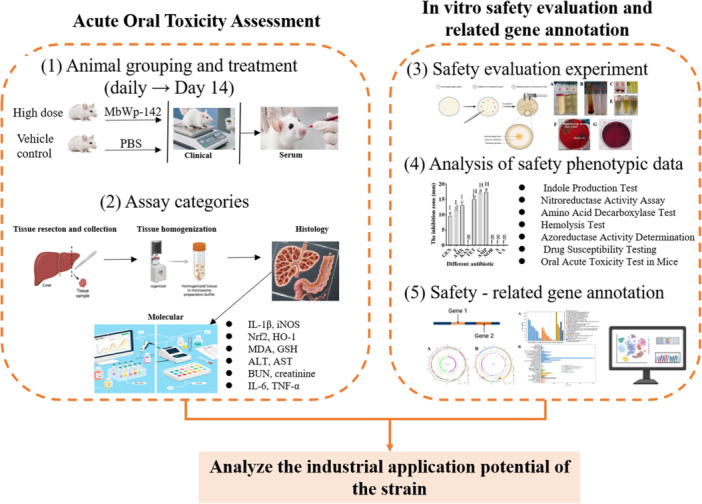

## 1 Introduction

Lactic acid bacteria (LAB) have long been integral to food fermentation and probiotic applications, celebrated for their functional versatility and generally recognized as safe (GRAS) status. Among these, *Weissella paramesenteroides* has emerged as a promising strain due to its dual potential: robust antibacterial activity against foodborne pathogens (e.g., *E. coli*, *Salmonella*) (Pabari et al. 2020; Pal and Ramana. 2009; Ashley et al. 2021; Papagianni and Papamichael 2011) and documented health benefits, including gut‐brain axis modulation and immune enhancement (Pabari et al. 2020; Prado et al. 2020; Sávio et al. 2020; Pal and Ramana 2010). For instance, *W. paramesenteroides* WpK4 has been shown to reduce anxiety‐like behaviors in murine models of colitis by improving gut barrier function (Sávio et al. 2020), while strains like DFR‐8 produce bacteriocins that inhibit pathogenic growth (Pal and Ramana. 2009). These attributes position *W. paramesenteroides* as a prime candidate for natural preservatives and functional food ingredients. (Fonseca et al. 2019; Amer et al. 2021; Carina et al. 2021).

Despite its potential, industrial translation of *W. paramesenteroides* remains constrained by a critical challenge: strain‐specific safety data. Recent studies have warned that some LAB isolates, including *Weissella* species, may harbor hidden risks such as hemolytic activity, biogenic amine production, or transferable antibiotic resistance genes (Jeong and Lee 2015). For example, Jeong and Lee. (2015) identified 12% of *Weissella* strains from kimchi samples exhibited β‐hemolysis, while Yadav and Shukla. (2022) reported that certain *W. paramesenteroides* strains produced trace amounts of histamine under suboptimal conditions. Such findings underscore the need for rigorous pre‐industrial safety profiling, particularly for strains isolated from novel sources.

The safety assessment of novel LAB strains is increasingly aligned with regulatory frameworks such as the European Food Safety Authority's (EFSA) Qualified Presumption of Safety (QPS) approach, which requires the absence of acquired antibiotic resistance genes and a clear demonstration of safety (EFSA 2021). While the genus *Weissella* is not yet included in the QPS list due to a lack of comprehensive safety data for all species, accumulating evidence suggests its clinical rarity and generally benign nature (Kamboj et al. 2015), underscoring the importance of strain‐specific evaluations like the present study.

Notably, *W. paramesenteroides* MW‐142 (hereinafter referred to as MW‐142)—originally isolated from traditional mulberry wine, a fermented food with centuries of safe human consumption— represents a unique case. While its ecological niche suggests historical safety, strain‐specific traits (e.g., metabolic pathways, genomic architecture) may vary significantly. Genomic comparisons of 40 *W. paramesenteroides* strains revealed extensive genetic diversity, including strain‐specific virulence factor annotations (Xing et al. 2023; Yadav and Shukla 2022), highlighting the danger of extrapolating safety profiles across isolates.

Our previous study optimized the high‐density cultivation of *W. paramesenteroides* MW‐142, achieving a cell density of 1.803 × 10^11^ CFU/mL (Huang et al. 2024), which laid the foundation for its large‐scale production. To translate this strain into practical use, comprehensive safety assessment is essential. Safety evaluation of LAB typically includes acute toxicity testing, hemolysis assessment, harmful metabolite analysis, and genomic screening for virulence and antibiotic resistance genes (Hu et al. 2023).

*W. paramesenteroides* MW‐142 was originally isolated from traditional mulberry wine, a fermented food with a long history of safe consumption. However, strain‐specific safety data are lacking. This study aimed to evaluate the biosafety of MW‐142 through a multi‐level approach, combining in vivo toxicity tests, in vitro biochemical assays, and whole‐genome analysis, to support its application in food and related industries.

Against this backdrop, this study addresses a critical void in the field: the comprehensive biosafety evaluation of *W. paramesenteroides* MW‐142. Leveraging a multi‐tiered approach— combining in vivo acute toxicity testing, in vitro hemolysis/metabolite assays, and whole‐genome screening for virulence/antibiotic resistance genes—we aim to: (1) Validate the safety of MW‐142 for oral consumption using murine models; (2) Characterize its potential to produce harmful metabolites (e.g., indole, biogenic amines); (3) Uncover genetic determinants of safety via comparative genomics.

This research not only fills a knowledge gap for a promising industrial strain but also establishes a benchmark safety evaluation framework for novel *Weissella* isolates, guiding their responsible integration into food, pharmaceutical, and biotechnological applications (Libonatti et al. 2019; Tozlu et al. 2019).

## 2 Materials and Methods

### 2.1 Strain and Culture Conditions

*Weissella paramesenteroides* MbWp‐142 (GDMCC 62249, NCBI accession: OM980714.1) was cultured in de Man, Rogosa, and Sharpe (MRS) medium at 37°C under anaerobic conditions. The strain is hereafter referred to as MW‐142 throughout this manuscript. Its complete genomic sequences are deposited in NCBI GenBank under accession numbers CP130559‐CP130560, and the BioProject accession number is PRJNA982423 (http://www.ncbi.nlm.nih.gov/bioproject/982423). The strain was activated for three generations before use (Huang et al. 2024).

### 2.2 Acute Oral Toxicity Assessment in SPF Mice

Specific pathogen‐free (SPF) Kunming mice (18–22 g, 10 males/10 females) were obtained from Zhejiang Weitong Lihua Experimental Animal Technology Co. Ltd. (Hangzhou, China), a CNAS‐certified supplier. The study design followed the OECD Guideline 420 for acute oral toxicity testing (OECD 2001). Upon arrival, mice were acclimatized for 7 days under controlled conditions (22 ± 2°C, 50 ± 10% humidity, 12 h light/dark cycle) with ad libitum access to sterile water and standard rodent chow. Mice were housed in polycarbonate cages (30 cm × 20 cm × 15 cm, 3–4 mice per cage of the same sex) with autoclaved corncob bedding replaced every 3 days. The SPF facility maintained ≥ 15 air changes per hour with HEPA filters (≥ 99.97% efficiency). All experimental procedures were performed by certified personnel, and humane endpoints were strictly observed. Euthanasia was performed by isoflurane anesthesia followed by cervical dislocation, in accordance with the AVMA Guidelines for the Euthanasia of Animals (American Veterinary Medical Association 2020).

#### 2.2.1 Dose Design and Administration

(1) Dose groups: High dose: 20.0 mL/kg BW (1.9 × 10^10^ CFU/kg, *n* = 10); Vehicle control: PBS (*n* = 10). Administration: Bacterial suspension (9.5 × 10^8^ CFU/mL) or PBS administered via oral gavage after 12 h fasting.

#### 2.2.2 Enhanced Toxicity Monitoring

(1) Clinical scoring: Daily assessment of 12 parameters (activity, fur condition, fecal consistency) using a 0–3 scoring system (Table 1) (Li 2004; FDA 2021). (2) Body weight kinetics: Measured daily to calculate growth rate (%) = [(final weight ‐ initial weight)/initial weight] × 100. (3) Subacute endpoints (Day 14): Serum biochemistry: Blood collected via retro‐orbital plexus for ALT, AST (liver), BUN, creatinine (kidney), and inflammatory cytokines (IL‐6, TNF‐α) using ELISA kits (R&D Systems). Organ index: Organ weight (g)/body weight (kg) for liver, kidney, spleen.

**Table 1:** Clinical scores of mice in acute oral toxicity test (mean ± SD, *n* = 10).

Parameter	(Day 1‐14)	Vehicle control (Day 1‐14)	*p* value
Activity level	2.8 ± 0.3	2.9 ± 0.2	0.312
Fur condition	2.7 ± 0.4	2.9 ± 0.1	0.156
Fecal consistency	2.6 ± 0.5	2.8 ± 0.3	0.248
Total score	8.1 ± 1.0	8.6 ± 0.5	0.089

*Note:* Scores are based on a 0–3 scale (0=normal, 3=severe abnormality). Statistical analysis by two‐way ANOVA with Bonferroni correction.

#### 2.2.3 Histopathological Analysis With Quantitative Scoring

Tissue processing: Liver, small intestine fixed in 10% formalin, embedded in paraffin, and sectioned at 5 μm. Quantitative scoring: A pathologist blinded to groups evaluated: Liver: Steatosis (0–4), portal inflammation (0–3); Colon: Villus atrophy (0–4), crypt hyperplasia (0–3). Scoring criteria adapted from the British Toxicology Society (BTS) guidelines.

#### 2.2.4 Molecular Safety Biomarkers

Gene expression analysis: qRT‐PCR for hepatic inflammatory genes (IL‐1β, iNOS) and renal stress markers (Nrf2, HO‐1) in liver/kidney tissues. Oxidative stress markers: Malondialdehyde (MDA) and glutathione (GSH) levels in liver/kidney homogenates (Cayman Chemical kits).

#### 2.2.5 Statistical Analysis

Two‐way ANOVA for body weight changes; Kruskal‐Wallis test for histopathological scores; Pearson correlation between serum biomarkers and organ indices. All analyses performed using GraphPad Prism 9, with *p* < 0.05 considered significant.

### 2.3 Harmful Metabolite Assays

(1) Indole production: MW‐142 was cultured in peptone water at 37°C for 72 h, and indole was detected using Kovacs' reagent (Boutroux et al. 2024). *Escherichia coli* ATCC 25922 served as the positive control, and a blank control (uninoculated medium) was included.

(2) Nitroreductase activity: MW‐142 was inoculated into nitrate broth and incubated at 37°C for 24 h. After incubation, 2–3 drops each of nitrate reduction reagents A and B were added. A color change to pink/red indicated nitrate reduction. For negative results, zinc powder was added to confirm the absence of false negatives (Hugo et al. 2009). *E. coli* ATCC 25922 was used as the positive control, and uninoculated medium as the blank control. All tests were performed in triplicate.

(3) Amino acid decarboxylase activity: MW‐142 was inoculated (3% v/v) into 5 mL of decarboxylase broth containing individual amino acids (lysine, arginine, ornithine) and overlaid with sterile liquid paraffin. Control tubes without amino acids were included. After incubation at 37°C for 24–72 h, a purple color (pH increase) indicated biogenic amine production; yellow color (acidic) indicated a negative result (Hussain 2011). All tests were performed in triplicate.

(4) Hemolysis Test: MW‐142 was streaked onto blood agar plates (containing 5% sheep blood) and incubated at 37°C for 48 h. Staphylococcus aureus ATCC 6538 was used as the positive control. Hemolytic zones around colonies were observed and recorded as α‐hemolysis (greenish zone), β‐hemolysis (clear zone), or γ‐hemolysis (no zone) (Bae et al. 2019).

(5) Azoreductase activity: MW‐142 was streaked onto MRS agar containing 5 mg/mL Direct Blue 71 and incubated at 37°C for 48–72 h. The formation of clear hydrolysis zones around colonies indicated positive azoreductase activity (Zhou et al. 2021).

### 2.4 Antibiotic Susceptibility Testing

Antibiotic susceptibility of MW‐142 was assessed using the Kirby‐Bauer disk diffusion method on MRS agar plates, following the protocol described by Lu et al. (2023) with minor modifications. Briefly, 100 µL of an overnight bacterial suspension (adjusted to approximately 1.0×10^9^ CFU/mL) was evenly spread onto MRS agar plates. Antibiotic disks (Oxoid, UK) containing the following antibiotics were placed on the agar surface using sterile forceps: gentamicin (10 µg), erythromycin (15 µg), chloramphenicol (30 µg), amoxicillin (25 µg), kanamycin (30 µg), tetracycline (30 µg), ampicillin (10 µg), norfloxacin (10 µg), vancomycin (30 µg), and streptomycin (10 µg). Disks were placed at least 24 mm apart (center to center) and at least 15 mm from the plate edge. After incubation at 37°C for 24 h under anaerobic conditions, the diameters of the inhibition zones were measured using a vernier caliper. All tests were performed in triplicate.

Since species‐specific clinical breakpoints for *Weissella* are not available in CLSI documents, the inhibition zone diameters were interpreted based on guidelines for infrequently isolated or fastidious bacteria (CLSI document M45, 3rd edition) and by comparison with published data for related LAB (EFSA 2012). For comparative purposes, we adopted the following descriptive criteria: Resistant (R)—no inhibition zone (0 mm); Low sensitivity (L)—zone diameter < 10 mm; Intermediate sensitivity (I)—zone diameter 10‐14 mm; High sensitivity (H)—zone diameter > 14 mm. These criteria are intended to provide a comparative overview of the strain's phenotypic resistance profile and are not clinical breakpoints.

### 2.5 Genomic Analysis of Safety‐Related Genes

The complete genome assembly and general functional annotation (COG, KEGG, CAZy) are described in detail in our companion paper (Huang et al. 2026). For this safety‐focused study, we specifically analyzed the genome against the VFDB and the Comprehensive Antibiotic Resistance Database (CARD). Genes related to virulence, toxin production, and antibiotic resistance were identified by BLAST (E‐value < 1 × 10^−10^, similarity ≥ 50%).

Genomic sequences of MW‐142 were queried against both databases using BLAST‐based algorithms. The absence of genes encoding key enzymes involved in harmful metabolite production, including indole synthase, nitroreductase, amino acid decarboxylase, and azoreductase, was systematically evaluated. This genomic‐level analysis provided molecular evidence supporting the phenotypic safety evaluation results, confirming that MW‐142 lacks genetic determinants associated with the biosynthesis of potentially hazardous compounds.

### 2.6 Analytical Methods

The cell density of MW‐142 was determined by the plate‐counting method. Serial dilutions of the culture broth were spread on MRS agar plates, and the plates were incubated at 30°C for 48 h under anaerobic conditions. The number of colonies was counted, and the cell density was expressed as colony ‐ forming units per milliliter (CFU/mL).

### 2.7 Statistical Analysis

All data were expressed as mean ± standard deviation (SD). Statistical analyses were performed using SPSS Statistics 22.0 (IBM, Armonk, NY, USA) and GraphPad Prism 9.0 (GraphPad, San Diego, CA, USA). Normality and homogeneity of variances were assessed using Shapiro–Wilk test and Levene's test, respectively. For comparisons between two groups, unpaired t‐test was used. For multiple group comparisons, one‐way ANOVA followed by Tukey's HSD post‐hoc test was applied when variances were homogeneous; Welch's ANOVA with Games–Howell post‐hoc test was used when variances were heterogeneous. Histopathological scores were analyzed using the Mann–Whitney U test. Outliers were identified using Grubbs' test (*α* = 0.05) and excluded only when attributable to non‐biological errors. Statistical significance was set at *p* < 0.05. Trends (0.05 ≤ *p* < 0.10) were noted for biologically relevant endpoints.

## 3 Results

### 3.1 General Clinical Observations and Body Weight Changes

**Figure 1:**
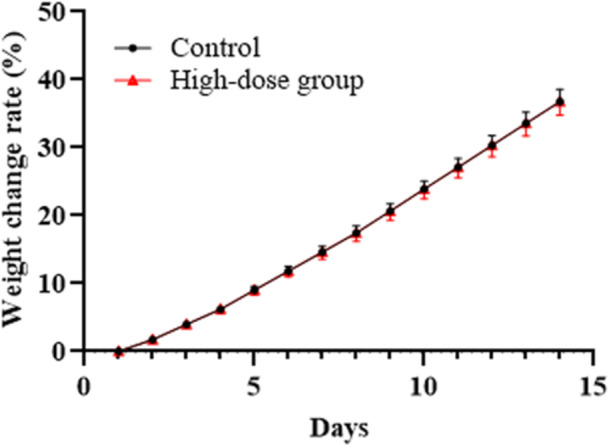
Body Weight Growth Rate of Mice During 14‐Day Observation. Data are presented as mean ± SD (*n* = 10 per group). The growth rate was calculated as [(final weight ‐ initial weight)/initial weight] × 100. No significant difference was observed between groups at any time point (*p* > 0.05, two‐way ANOVA).

Over the 14‐day observation period, no overt toxic signs (e.g., lethargy, abnormal posture, diarrhea) were noted in either group. Clinical scoring of 12 parameters (activity, fur condition, fecal consistency, etc.) showed no significant differences, with total scores of 8.1 ± 1.0 (high‐dose) versus 8.6 ± 0.5 (control; *p* = 0.089, Table 1). Body weight gain trends were comparable, with 14‐day growth rates of 24.8 ± 3.2% and 26.5 ± 2.8% in high‐dose and control groups, respectively (*p* > 0.05, Figure 1), indicating MW‐142 did not impair growth.

### 3.2 Serum Biochemistry and Inflammatory Cytokines

Day 14 serum analyses revealed similar hepatic (ALT: 25.2 ± 4.1 vs. 22.8 ± 3.5 U/L; AST: 62.5 ± 7.3 vs. 68.3 ± 6.8 U/L) and renal (BUN, creatinine) marker levels between groups (*p* > 0.05, Table 2). ELISA‐detected IL‐6 (12.5 ± 1.8 vs. 11.2 ± 1.5 pg/mL) and TNF‐α (8.8 ± 1.2 vs. 7.9 ± 1.0 pg/mL) showed marginal, non‐significant elevations in the high‐dose group (*p* = 0.093 and 0.134, respectively). This slight increase in IL‐6, while not statistically significant, may reflect a mild, transient immune stimulation commonly observed with probiotic consumption, rather than an adverse inflammatory response.

**Table 2:** Serum Biochemistry and Inflammatory Cytokines in Mice (Day 14, Mean ± SD, n = 10).

Index	High‐dose group	Vehicle control	*p* value
Liver function			
ALT (U/L)	25.2 ± 4.1	22.8 ± 3.5	0.102
AST (U/L)	62.5 ± 7.3	68.3 ± 6.8	0.167
Kidney function			
BUN (mmol/L)	5.2 ± 0.5	4.9 ± 0.4	0.215
Creatinine (μmol/L)	28.6 ± 3.2	26.9 ± 2.8	0.189
Inflammatory factors			
IL‐6 (pg/mL)	12.5 ± 1.8	11.2 ± 1.5	0.093
TNF‐α (pg/mL)	8.8 ± 1.2	7.9 ± 1.0	0.134

*Note:* Statistical analysis by unpaired t‐test. ALT: alanine transaminase; AST: aspartate transaminase; BUN: blood urea nitrogen

### 3.3 Organ Indexes

**Figure 2:**
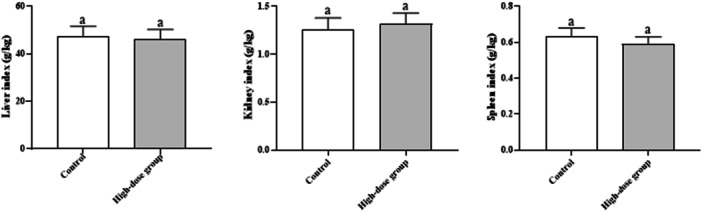
Organ Indexes of Mice (Day 14). Data are presented as mean ± SD (*n* = 10 per group). Organ index = organ weight (g)/body weight (kg). No significant differences were observed between groups (*p* > 0.05, unpaired t‐test).

No significant differences in liver (47.36 ± 4.21 vs. 46.15 ± 4.04 g/kg), kidney (1.26 ± 0.12 vs. 1.32 ± 0.11 g/kg), or spleen (0.63 ± 0.05 vs. 0.59 ± 0.04 g/kg) indexes were observed on day 14 (*p* > 0.05, Figure 2), ruling out organ‐specific toxicity.

### 3.4 Histopathological Findings in Liver and Colon

Histological examination revealed no severe abnormalities. Mild hepatic steatosis (0.5 ± 0.2 vs. 0.3 ± 0.1) and portal inflammation (0.2 ± 0.1 vs. 0.1 ± 0.0) in the high‐dose group were comparable to controls (*p* > 0.05). Colon scores for villus atrophy (0.3 ± 0.1 vs. 0.2 ± 0.1) and crypt hyperplasia (0.2 ± 0.1 vs. 0.1 ± 0.0) also showed no significant differences (*p* > 0.05, Table 3), with all changes within normal physiological ranges.

**Table 3:** Histopathological Scores of Liver and Colon (Day 14, Mean ± SD, n = 5).

Tissue	Parameter	High‐dose group	Vehicle control	*p* value
Liver	Steatosis (0‐4)	0.5 ± 0.2	0.3 ± 0.1	0.124
	Portal inflammation (0‐3)	0.2 ± 0.1	0.1 ± 0.0	0.267
Colon	Villus atrophy (0‐4)	0.3 ± 0.1	0.2 ± 0.1	0.318
	Crypt hyperplasia (0‐3)	0.2 ± 0.1	0.1 ± 0.0	0.291

*Note:* Scores were evaluated by a blinded pathologist. Statistical analysis by Mann‐Whitney U test

### 3.5 Molecular Biomarkers of Inflammation and Oxidative Stress

**Figure 3:**
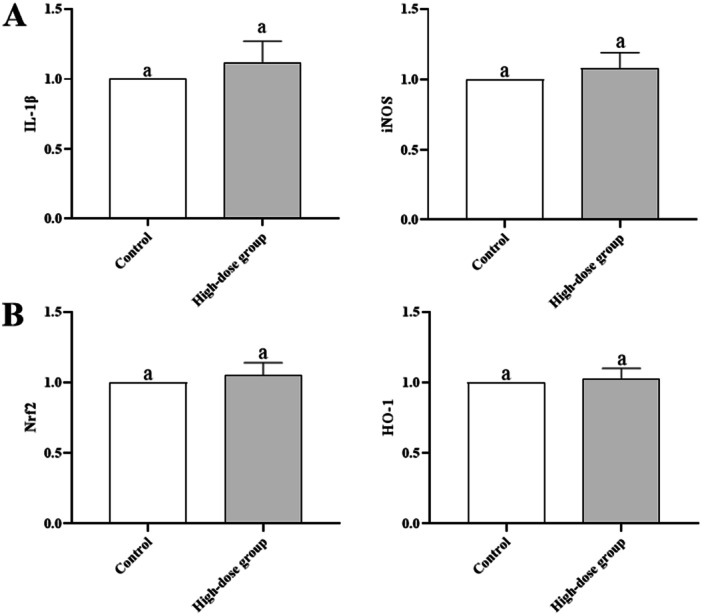
Relative Expression of Inflammatory Genes and Stress Markers (Day 14). Data are presented as mean ± SD (*n* = 6 per group). Relative expression was normalized to β‐actin using the 2^(‐ΔΔCt) method. A: Hepatic IL‐1β and iNOS; B: Renal Nrf2 and HO‐1. No significant differences were observed between groups (*p* > 0.05, unpaired t‐test).

**Figure 4:**
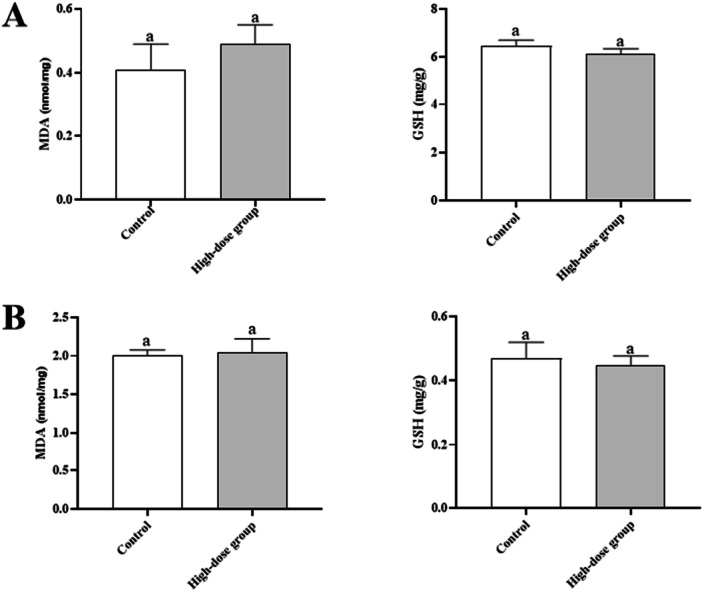
Oxidative Stress Markers in Liver and Kidney (Day 14). Data are presented as mean ± SD (*n* = 6 per group). A: Hepatic MDA and GSH; B: Renal MDA and GSH. No significant differences were observed between groups (*p* > 0.05, unpaired t‐test).

qRT‐PCR analysis showed no upregulation of hepatic IL‐1β (1.12 ± 0.15), iNOS (1.08 ± 0.11), or renal Nrf2 (1.05 ± 0.09), HO‐1 (1.03 ± 0.07) in the high‐dose group (*p* > 0.05, Figure 3). Hepatic and renal MDA/GSH levels were similar between groups (*p* > 0.05, Figure 4), indicating no oxidative stress.

Collectively, acute oral administration of MW‐142 at 1.9 × 10^10^ CFU/kg does not induce systemic, hepatic, renal, or intestinal toxicity in SPF Kunming mice.

### 3.6 Harmful Metabolite Assays

**Figure 5:**
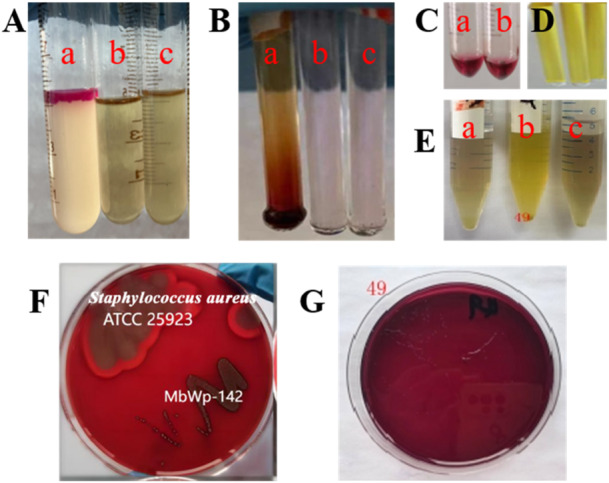
Harmful metabolite analysis. Results of indole production test (A), nitroreductase activity assay (B). In both A and B, (a‐c) represent the *Escherichia coli* positive control group, the blank control group, and MW‐142 in sequence. C is the result diagram of the recheck of the nitroreductase test, and in C (a‐b) represent the blank control group and the experimental group. D shows the four strains to be tested cultured in ornithine‐containing biochemical tubes. E is the result of the amino acid reductase test. E (a‐c) are the blank culture solutions, the culture solutions containing lysine, and the culture solutions containing arginine. F and G are the result diagrams of the hemolysis test and the azoreductase activity test, respectively. The performance of MW‐142 in each test is intuitively shown by comparison with the control strain to evaluate its safety.

Indole production: In the indole production test, the ether layer of the MW‐142 culture remained colorless (Figure 5A), contrasting with the red coloration observed in the *E. coli* positive control. This negative result indicates that MW‐142 lacks tryptophanase activity, the enzyme responsible for degrading tryptophan to produce indole (Boutroux et al. 2024), thereby confirming its inability to produce this potential harmful metabolite.

Nitroreductase activity: No color change was observed after adding nitrate reagents, and rechecking with zinc granules confirmed negative results. For the nitroreductase activity assay, all tested strains showed a pink coloration after adding nitrate reduction reagents (Figure 5B and C), indicating negative results. Upon adding zinc granules for re‐examination, the solution turned red, confirming that the initial negative results were not false positives. These findings suggested MW‐142 strain either lacked nitrate reductase or had low nitrate reductase activity.

Amino acid decarboxylase activity: Cultures showed no pH increase, indicating no biogenic amine production. In the amino acid decarboxylase test, the culture solutions of MW‐142 strain, after incubation in media containing different amino acids, exhibited a yellow color similar to that of the blank control group (media without amino acids) (Figure 5 D), indicating negative results. This demonstrated that the MW‐142 strain either lacked amino acid decarboxylase or had low amino acid decarboxylase activity.

Hemolysis Test: As shown in Figure 5 F, on blood agar plates, *S. aureus* ATCC 25923 (positive control) produced clear, well‐defined zones of β‐hemolysis surrounding its colonies, indicating complete lysis of red blood cells. In contrast, MW‐142 colonies appeared as creamy‐white, opaque colonies with no surrounding clearing zone, consistent with γ‐hemolysis (non‐hemolytic activity).

Azoreductase activity: No hydrolysis zones were detected on Direct Blue 71‐containing agar, suggesting low azoreductase activity. The azoreductase activity assay results (Figure 5 G) showed that the media of MW‐142 strain remained purplish‐red without the formation of hydrolysis zones, indicating negative results. This indicated that MW‐142 strain either lacked azoreductase or had low azoreductase activity. Additionally, the slow growth and small colony size of the tested strains on the media suggested that Direct Blue 71 might inhibit or be toxic to these strains.

### 3.7 Antibiotic Susceptibility

**Figure 6:**
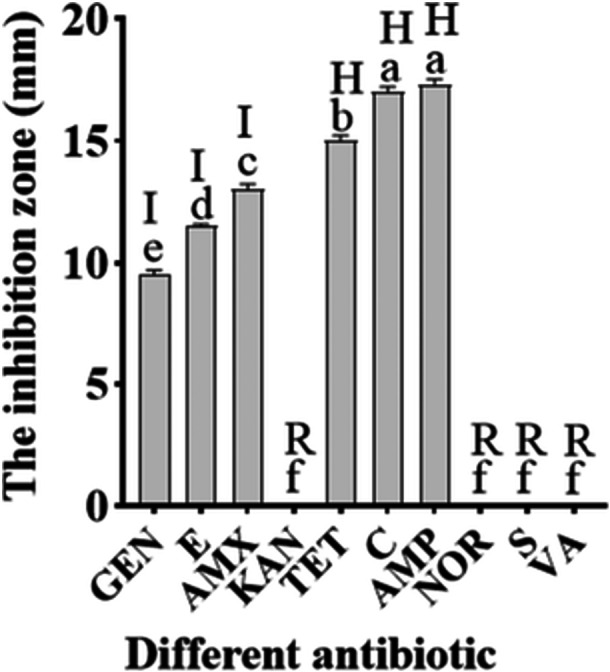
Results of the antibiotic susceptibility test of MW‐142 using the Kirby‐Bauer disc diffusion method, showing the susceptibility of the strain to ten antibiotics (tetracycline, gentamicin, chloramphenicol, streptomycin, amoxicillin, ampicillin, kanamycin, erythromycin, norfloxacin, vancomycin). *Note*: Inhibition zone diameter: Resistant (R): 0 mm; Low sensitivity (L): < 10 mm; Intermediate sensitivity (I): 10‐14 mm; High sensitivity (H): > 14 mm.

The antibiotic susceptibility profile of MW‐142 was determined by the disk diffusion method, and the inhibition zone diameters (mean ± SD of triplicate measurements) are presented in Table 4. As shown in Figure 6, MW‐142 exhibited high sensitivity (zone > 14 mm, classified as H according to the criteria described in Section 2.4) to tetracycline (15.00 ± 0.50 mm), chloramphenicol (17.00 ± 0.82 mm), and ampicillin (17.33 ± 0.58 mm). Intermediate sensitivity (zone 10–14 mm, classified as I) was observed for erythromycin (11.50 ± 0.71 mm) and amoxicillin (13.00 ± 0.50 mm). Low sensitivity (zone < 10 mm, classified as L) was observed for gentamicin (9.50 ± 0.58 mm). The strain showed resistance (no inhibition zone, classified as R) to kanamycin (0 mm), norfloxacin (0 mm), streptomycin (0 mm), and vancomycin (0 mm) (Table 4). These results indicate that MW‐142 is susceptible to multiple clinically relevant antibiotics but displays resistance to several others, which is common among *Weissella* and related LAB genera (Toropov et al. 2020).

**Table 4:** Inhibition zone diameters of antibiotics against MW‐142.

Antibiotic	Disk content (µg)	Inhibition zone (mm)	Classification
Tetracycline (TET)	30	15.00 ± 0.50	H
Chloramphenicol (C)	30	17.00 ± 0.82	H
Ampicillin (AMP)	10	17.33 ± 0.58	H
Erythromycin (E)	15	11.50 ± 0.71	I
Amoxicillin (AMX)	25	13.00 ± 0.50	I
Gentamicin (GEN)	10	9.50 ± 0.58	L
Kanamycin (KAN)	30	0	R
Norfloxacin (NOR)	10	0	R
Streptomycin (S)	10	0	R
Vancomycin (VA)	30	0	R

*Note:* Values are the mean of three independent replicates ± standard deviation (SD). Classification based on criteria described in Section 2.4: H (High sensitivity) > 14 mm; I (Intermediate sensitivity) 10–14 mm; L (Low sensitivity) < 10 mm; R (Resistant) 0 mm.

Genomic analysis using the CARD database identified several putative antibiotic resistance genes, including *mdeA*, *lmrP*, *rfbB*, and *acpP* (Supplementary Table S1). Importantly, none of these genes were found to be associated with mobile genetic elements such as transposons or plasmids, suggesting a low risk of horizontal transfer (Pursey et al. 2021). The phenotypic resistance to vancomycin is consistent with the presence of intrinsic resistance mechanisms commonly found in *Weissella* (e.g., modified cell wall precursors) and does not involve acquired resistance genes.

### 3.8 Genomic Analysis of Safety‐Related Genes

Genomic screening against the virulence factor database (VFDB) identified 83 putative virulence‐associated genes in MW‐142, accounting for 4.29% of its genome (Supporting Information S1: Table S2). However, over half of these genes shared < 50% sequence similarity with known virulence factors in VFDB. Notably, key toxin‐encoding genes (e.g., *hlyD*, *hlyIII*) were absent, as were genes for indole synthase, nitroreductase, and amino acid decarboxylase–enzymes linked to potential toxic metabolite production.

Antibiotic resistance gene (ARG) analysis using the Comprehensive Antibiotic Resistance Database (CARD) identified several putative antibiotic resistance genes, including *mdeA*, *mdtG*, and *lmrP* (Supporting Information S1: Table S1). None of these ARGs were flanked by transposons or mobile genetic elements, consistent with phenotypic susceptibility data showing low transfer risk (Saroj and Gupta 2020). Further annotation identified stress‐response genes: *rfbB*, *rmlB*, *rffG* (MW‐142AGL000153, associated with vancomycin, streptomycin, and amikacin tolerance) and *acpP* (MW‐142AGL000594 and MW‐142AGL001230, linked to antibiotic synthesis), aligning with phenotypic observations of high tolerance to streptomycin, kanamycin, norfloxacin, and vancomycin, moderate tolerance to gentamicin, and weak tolerance to erythromycin, amoxicillin, and tetracycline.

KEGG and COG analyses detected 16 transposon‐related genes but no evidence of ARG integration into mobile elements. No classic resistance determinants (e.g., *tetM*, *ermB*, *bla*) were found, supporting the strain's low risk of horizontal resistance gene transfer (Handajani et al. 2023; Pursey et al. 2021).

### 3.9 Activity of Harmful Enzymes

When searching for core terms related to the activities of harmful enzymes (such as N‐acetyl‐β‐glucosaminidase, β‐glucosidase, and β‐glucuronidase) in the results of general functional annotation, no relevant enzymes were found. Combined with the experimental verification results in Section 3.6, it suggests that MW‐142 has a very high level of safety.

## 4 Discussion

The safety assessment of LAB intended for food and probiotic applications is a fundamental prerequisite for their commercial development (Rodriguez et al. 2023). This study provides a comprehensive safety evaluation of *Weissella paramesenteroides* MW‐142, a strain previously optimized for high‐density cultivation (Huang et al. 2024), through an integrated approach combining in vivo toxicity testing, in vitro biochemical assays, and whole‐genome sequencing. The collective findings demonstrate that MW‐142 possesses a robust safety profile supporting its potential use in food fermentation and probiotic products.

The absence of acute oral toxicity in mice represents the most fundamental safety indicator. Administration of MW‐142 at a high dose (1.9 × 10^10^ CFU/kg body weight) produced no mortality, clinical signs of distress, or adverse effects on body weight gain, organ indices, or serum biochemical parameters over the 14‐day observation period. Histopathological examination of liver and colon tissues revealed no structural abnormalities, and molecular biomarkers showed no evidence of inflammation (IL‐1β, iNOS) or oxidative stress (Nrf2, HO‐1, MDA, GSH). These in vivo findings are consistent with safety evaluations of other prospective *Weissella* probiotics, including *W. paramesenteroides* MYPS5.1, which also demonstrated no toxicity in murine models (Yadav and Shukla. 2022). The marginal, non‐significant increase in IL‐6 observed in the high‐dose group falls within the normal physiological range and may reflect a mild, transient immune stimulation commonly associated with probiotic consumption rather than an adverse inflammatory response (Vinderola and Reinheimer 2020).

Phenotypic safety assays further corroborated the in vivo observations. The non‐hemolytic activity (γ‐hemolysis) of MW‐142 is particularly noteworthy, as hemolytic potential has been documented in some LAB isolates. Jeong and Lee. (2015) reported that 12% of *Weissella* strains isolated from kimchi exhibited β‐hemolytic activity, highlighting the strain‐to‐strain variability within this genus and underscoring the importance of strain‐specific safety assessment. The absence of amino acid decarboxylase activity in MW‐142 distinguishes it from certain *W. paramesenteroides* strains that have been shown to produce trace amounts of biogenic amines under suboptimal conditions (Yadav and Shukla 2022). Biogenic amine accumulation in fermented foods remains a food safety concern, and the inability of MW‐142 to decarboxylate amino acids minimizes this risk (Zapaśnik et al. 2022). Additionally, negative results for indole production, nitroreductase activity, and azoreductase activity further reduce concerns regarding the potential in situ generation of toxic or carcinogenic metabolites (Hugo et al. 2009; Tripathi et al. 2022).

Antibiotic resistance profiling of probiotic candidates requires careful evaluation to ensure that resistance determinants do not pose a risk of horizontal transfer to pathogenic bacteria. Phenotypically, MW‐142 demonstrated high sensitivity to tetracycline, chloramphenicol, and ampicillin; intermediate sensitivity to erythromycin and amoxicillin; low sensitivity to gentamicin; and resistance to kanamycin, norfloxacin, streptomycin, and vancomycin. Resistance to vancomycin is commonly observed in *Weissella* and many other LAB genera and is typically intrinsic and non‐transferable, often mediated by modified cell wall precursors (Toropov et al. 2020). Genomic analysis using the CARD database identified several putative antibiotic resistance genes, including *mdeA*, *lmrP*, *rfbB*, and *acpP*. Critically, none of these genes were found to be associated with mobile genetic elements such as transposons or plasmids, and key transferable resistance determinants (e.g., tetM, ermB, bla) were absent. The absence of mobile genetic elements adjacent to resistance genes substantially minimizes the risk of resistance dissemination, a key concern in probiotic safety assessment (Pursey et al. 2021; Chaichana et al. 2025).

Genomic mining for virulence determinants revealed 83 putative virulence‐associated genes, a finding common in LAB genomes (Liu et al. 2022). However, over half (47/83) of these genes exhibited less than 50% sequence similarity to known virulence factors in the VFDB, suggesting they may encode proteins with alternative primary functions (e.g., stress response, capsule synthesis, exopolysaccharide production) rather than true pathogenic potential. Importantly, genes encoding classical toxins such as hemolysins and enterotoxins were absent or non‐functional. The presence of capsule and exopolysaccharide biosynthesis genes is consistent with their roles in stress tolerance and probiotic‐host interactions, rather than virulence per se (Xing et al. 2023).

Within the framework of the EFSA Qualified Presumption of Safety (QPS) approach, microbial strains intended for use in food and feed must demonstrate the absence of acquired antibiotic resistance genes and provide clear evidence of safety (EFSA 2021). Although the genus *Weissella* is not yet included in the QPS list due to insufficient strain‐specific data across all species, accumulating epidemiological evidence suggests its clinical rarity and generally benign nature (Kamboj et al. 2015). The present study addresses this data gap for MW‐142 by providing comprehensive evidence that meets the QPS criteria: no acquired antibiotic resistance genes associated with mobile elements, no toxigenic potential, and no adverse effects in an in vivo model.

### 4.1 Limitations and Future Perspectives

Several limitations of this study should be acknowledged. First, the acute toxicity test, while essential for initial safety screening, does not capture potential long‐term effects. A sub‐chronic (90‐day) toxicity study in rodents would provide additional assurance regarding the safety of prolonged exposure. Second, although genomic analysis revealed no association between resistance genes and mobile elements, conjugation experiments could provide definitive experimental proof of non‐transferability. Third, the disk diffusion method employed for antibiotic susceptibility testing, while adequate for comparative phenotypic profiling, is less quantitative than the LSM broth microdilution method recommended by EFSA; future studies should adopt this gold‐standard method for precise MIC determination (EFSA 2018). Fourth, while MW‐142 demonstrated a favorable safety profile in healthy mice, its safety in immunocompromised or vulnerable populations remains to be established.

Future research directions should include: (1) long‐term toxicity evaluation in animal models to confirm safety for chronic consumption; (2) experimental validation of antibiotic resistance gene non‐transferability through conjugation assays; (3) investigation of the strain's interaction with the gut microbiota and its immunomodulatory effects in controlled human trials; (4) assessment of its efficacy and safety as a biopreservative in relevant food matrices; and (5) clinical trials in target populations to evaluate both probiotic efficacy and safety under real‐world conditions. Such studies will bridge the gap between preclinical safety assessment and commercial application.

In conclusion, this comprehensive safety evaluation of *W. paramesenteroides* MW‐142, integrating in vivo, phenotypic, and genomic approaches, establishes its suitability as a safe candidate for further development in food and pharmaceutical applications. Together with our companion paper characterizing its probiotic mechanisms (Huang et al. 2026, The Microbe), these findings provide a complete safety and functionality profile supporting the industrial potential of this strain.

## 5 Conclusion

This study presents the first comprehensive biosafety evaluation of *Weissella paramesenteroides* MW‐142, a strain isolated from traditionally fermented mulberry wine with proven high‐density cultivation potential. Through an integrated approach combining in vivo acute oral toxicity testing in mice, a full panel of in vitro biochemical assays for harmful metabolites, and a thorough in silico genomic screening using updated databases, we have established a robust safety profile for this strain. The key findings–no adverse effects in the animal model, non‐hemolytic activity, absence of biogenic amine production, and the identification of only intrinsic, non‐mobile antibiotic resistance genes—collectively position MW‐142 as a safe candidate for industrial applications. While these results are highly promising, they warrant confirmation in long‐term toxicity studies and experimental validation of gene non‐transferability. This work not only supports the commercial development of MW‐142 but also provides a rigorous methodological benchmark for the safety assessment of novel LAB isolates intended for use in food, pharmaceuticals, and biotechnology.

## 6 Consent for Publication

We, the undersigned author(s), hereby grant permission for the publication of the manuscript entitled [Safety Evaluation of *Weissella paramesenteroides* MW‐142 Isolated from Traditional Fermented Mulberry Wine] in the intended journal. We confirm that this manuscript represents original work, has not been previously published (excluding preprints or conference proceedings), and is not under consideration for publication elsewhere. We understand that the manuscript will be published online and potentially in print, and we agree to transfer the copyright of the manuscript to the journal. We declare that all authors have contributed significantly to the study and manuscript preparation and have reviewed and approved the final version of the manuscript.

## Author Contributions

**Juan Huang:** conceptualization (lead), investigation (lead), writing – original draft (lead), writing – review and editing (equal). **Yan‐yan Huang:** conceptualization (supporting), methodology (lead), Writing – review and editing (equal). **Dong‐mei Liu:** conceptualization (lead), funding acquisition (lead), supervision (lead), writing – review and editing (equal). **Qing‐bo Yao:** methodology (supporting), writing – review and editing (equal). **Jun Tang:** investigation (supporting), writing – review and editing (equal). **Su‐ping Zeng:** investigation (supporting), writing – review and editing (equal). **Liu‐jun Liu:** investigation (supporting), writing – review and editing (equal).

## Ethics Statement

All animal procedures in this study were approved by the Experimental Animal Ethics Committee of Foshan University (Approval No.: SYXK 2020‐0235) and conducted in accordance with the Regulations for the Administration of Experimental Animals of China (State Council of the People's Republic of China 2017) and the Guide for the Care and Use of Laboratory Animals (National Institutes of Health 2011). Every effort was made to minimize animal suffering and to reduce the number of animals used, following the 3Rs principle (Replacement, Reduction, Refinement).

## Conflicts of Interest

The authors declare no conflicts of interest.

## Supporting information


**Table S1:** Putative antibiotic resistance genes identified in W. paramesenteroides MW‐142 by CARD analysis.
**Table S2:** Analyzing the function of these virulence‐related factors of W. paramesenteroides MW‐142.

## Data Availability

The whole‐genome sequencing data for *Weissella paramesenteroides* MW‐142 have been deposited in NCBI GenBank under accession numbers CP130559‐CP130560. The associated BioProject is PRJNA982423 (http://www.ncbi.nlm.nih.gov/bioproject/982423). A comprehensive analysis of the genomic features related to carbohydrate metabolism, stress response, and other probiotic mechanisms of this strain has been published separately in our companion paper (Huang et al. 2026, *The Microbe* 10:100671). All other data generated or analyzed during this study are included in this published article and its supplementary information files.The data that support the findings of this study are openly available in GenBank at https://submit.ncbi.nlm.nih.gov/subs/wgs/SUB13493371/overview, reference number CP130559‐CP130560.
