# Supplementary material for: Safety Evaluation of Weissella paramesenteroides MW‐142 Isolated From Traditional Fermented Mulberry Wine
Source: Microbiologyopen. 2026 May 11;15(3):e70302. doi: 10.1002/mbo3.70302 (PMC13158872; doi:10.1002/mbo3.70302)
Supplement: Supplementary file 1 — Table S1: Putative antibiotic resistance genes identified in W. paramesenteroides MW‐142 by CARD analysis. Table S2: Analyzing the function of these virulence‐related factors of W. paramesenteroides MW‐142. [file MBO3-15-e70302-s001.docx]

**Supplementary Table S1** **|** Putative antibiotic resistance genes identified in *W. paramesenteroides* MW-142 by CARD analysis

| Gene ID | Gene name | Description / Product | CARD match (ARO term) | Identity (%) | Query coverage (%) | E-value | Flanked by mobile elements |
| --- | --- | --- | --- | --- | --- | --- | --- |
| MW-142AGL000685 | *mdeA* | MFS transporter, DHA2 family, multidrug resistance protein | K18936 (mdeA) | 62.5 | 99.6 | 1.1E-182 | Not detected |
| MW-142AGL001107 | *mdtG* | MFS transporter, DHA1 family, multidrug resistance protein | K08161 (mdtG) | 61.5 | 100 | 2E-133 | Not detected |
| MW-142AGL001638 | *mdeA* | MFS transporter, DHA2 family, multidrug resistance protein | K18936 (mdeA) | 67.8 | 99.2 | 1.8E-181 | Not detected |
| MW-142AGL001666 | *lmrP* | MFS transporter, DHA1 family, multidrug resistance protein B | K08152 (lmrP) | 52.5 | 99.8 | 2.8E-122 | Not detected |

**Supplementary TABLE S2 |** Analyzing the function of these virulence-related factors of *W. paramesenteroides* MW-142

| Gene_id | Identity/% | E_value | Type | Annotion | Description | Classification |
| --- | --- | --- | --- | --- | --- | --- |
| MW-142AGL000106 | 48.4 | 7.40E-128 | Predicted | katA | catalase | Catalase |
| MW-142AGL000137 | 50.7 | 3.10E-36 | Predicted | oppF | oligopeptideABCtransporter,permeasecomponent | Capsule |
| MW-142AGL000153 | 66.4 | 3.50E-140 | Predicted | STER_1222 | dTDP-D-glucose4,6-dehydratase | Capsule |
| MW-142AGL000159 | 45.8 | 3.60E-58 | Predicted | EFD32_0902 | sortasefamilyprotein | Ebppili |
| MW-142AGL000179 | 51.4 | 6.70E-97 | Predicted | sugC | carbohydrateABCtransporterATP-bindingprotein,CUT1family | Trehalose-recyclingABCtransporter |
| MW-142AGL000217 | 64.7 | 1.70E-177 | Predicted | KPHS_35590 | 6-phosphogluconatedehydrogenase | Capsule |
| MW-142AGL000227 | 40.5 | 2.50E-88 | Predicted | FN3523_0439 | N-acetylglucosamine-1-phosphateuridyltransferase/glucosamine-1-phosphateN-acetyltransferase | LPS |
| MW-142AGL000247 | 50 | 3.90E-58 | Predicted | regX3 | twocomponenttranscriptionalregulator | RegX3 |
| MW-142AGL000252 | 49.1 | 7.90E-99 | Predicted | htrA/degP | serineprotease | Serineprotease |
| MW-142AGL000276 | 40.8 | 7.00E-114 | Predicted | oppA | oligopeptideABCtransportersubstrate-bindingprotein | Oligopeptide-bindingprotein |
| MW-142AGL000280 | 46.4 | 2.70E-38 | Predicted | oppF | oligopeptideABCtransporter,permeasecomponent | Capsule |
| MW-142AGL000301 | 40.4 | 1.40E-34 | Predicted | sugC | sugar ABC transporter | Trehalose-recycling ABC transporter |
| MW-142AGL000342 | 60.1 | 6.50E-86 | Predicted | *uppS* | undecaprenyl diphosphate synthase | Capsule |
| MW-142AGL000343 | 49.1 | 1.00E-67 | Verified | *cpsB* | phosphatidate cytidylyltransferase | Capsule |
| MW-142AGL000428 | 40.4 | 8.60E-34 | Predicted | *regX3* | response regulator with CheY-like receiver domain and winged-helix DNA-binding domain | RegX3 |
| MW-142AGL000442 | 40.9 | 8.30E-55 | Predicted | *sugC* | Probable sugar ABC transporter, ATP-binding protein SugC | Trehalose-recycling ABC transporter |
| MW-142AGL000446 | 55.6 | 3.80E-62 | Predicted | *slrA* | peptidyl-prolyl cis-trans isomerase, cyclophilin-type | Streptococcal lipoprotein rotamase A |
| MW-142AGL000453 | 72.4 | 1.10E-118 | Predicted | *SMU.322c* | glucose-1-phosphate uridylyltransferase | Capsule |
| MW-142AGL000463 | 69.2 | 1.00E-171 | Predicted | *eno* | phosphopyruvate hydratase | Streptococcal enolase |
| MW-142AGL000468 | 40 | 8.90E-37 | Predicted | *sugC* | maltodextrin import ATP-binding protein MsmX | Trehalose-recycling ABC transporter |
| MW-142AGL000480 | 41.9 | 6.30E-97 | Predicted | *mrsA/glmM* | phosphoglucosamine mutase | Exopolysaccharide |
| MW-142AGL000532 | 55.3 | 1.30E-99 | Predicted | *plr/gapA* | glyceraldehyde-3-phosphate dehydrogenase, type I | Streptococcal plasmin receptor/GAPDH |
| MW-142AGL000538 | 41 | 3.40E-38 | Predicted | *sugC* | ABC transporter ATP-binding protein | Trehalose-recycling ABC transporter |
| MW-142AGL000591 | 42.3 | 4.30E-23 | Predicted | *fabZ* |  | 3R |
| MW-142AGL000597 | 44.9 | 1.00E-53 | Predicted | *flmH* | 3-oxoacyl-ACP reductase | Polar flagella |
| MW-142AGL000600 | 41.5 | 5.90E-25 | Predicted | *fabZ* |  | 3R |
| MW-142AGL000620 | 42.8 | 4.00E-93 | Predicted | *lysA* | diaminopimelate decarboxylase | Lysine synthesis |
| MW-142AGL000700 | 42.5 | 2.00E-92 | Verified | *cpsJ* | ABC transporter, ATP-binding protein | Capsule |
| MW-142AGL000720 | 48.2 | 7.40E-96 | Predicted | *sugC* | carbohydrate ABC transporter ATP-binding protein, CUT1 family | Trehalose-recycling ABC transporter |
| MW-142AGL000758 | 53.2 | 1.50E-53 | Predicted | *epsE* | exopolysaccharide biosynthesis protein | Capsule |
| MW-142AGL000762 | 71.7 | 2.90E-167 | Verified | *cpsI* | UDP-galactopyranose mutase | Capsule |
| MW-142AGL000765 | 41.2 | 4.80E-103 | Predicted | *STER_1057* | Polysaccharide Transporter, PST family | Capsule |
| MW-142AGL000766 | 42.1 | 8.50E-61 | Verified | *gtrB* | bactoprenol glucosyl transferase | LPS |
| MW-142AGL000773 | 56.6 | 1.90E-189 | Predicted | *CT396* | molecular chaperone DnaK | MOMP |
| MW-142AGL000783 | 43.5 | 3.90E-11 | Verified | *cap8J* | capsular polysaccharide synthesis enzyme Cap8J | Capsule |
| MW-142AGL000784 | 46.4 | 1.20E-24 | Predicted | *mgtC* | MgtC/SapB transporter | Magnesium transport |
| MW-142AGL000805 | 40.7 | 4.20E-154 | Predicted | *relA* |  | P |
| MW-142AGL000814 | 45 | 1.00E-33 | Predicted | *scpB* | segregation and condensation protein B | Fibronectin-binding protein |
| MW-142AGL000823 | 42 | 1.00E-14 | Predicted | *ML1683* | histone-like protein | histone-like protein |
| MW-142AGL000840 | 40.3 | 3.50E-51 | Predicted | *cmaA2* | Cyclopropane-fatty-acyl-phospholipid synthase | Mycolic acid trans cyclopropane synthetase |
| MW-142AGL000849 | 41 | 9.80E-113 | Predicted | *oppA* | oligopeptide ABC transporter substrate-binding protein | Oligopeptide-binding protein |
| MW-142AGL000958 | 70.6 | 2.10E-88 | Predicted | *lisR* | two-component response regulator | LisR/LisK |
| MW-142AGL000988 | 49.6 | 2.90E-56 | Predicted | *regX3* | Sensory transduction protein RegX3 | RegX3 |
| MW-142AGL000991 | 55.6 | 2.40E-105 | Predicted | *sigA/rpoV* | RNA polymerase sigma factor rpoD | Sigma A |
| MW-142AGL001019 | 44.8 | 1.20E-76 | Predicted | *pdhB* | pyruvate dehydrogenase E1 component, beta subunit | PDH-B |
| MW-142AGL001021 | 41.8 | 7.70E-68 | Predicted | *lplA1* | lipoyltransferase and lipoate-protein ligase family protein | Lipoate protein ligase A1 |
| MW-142AGL001040 | 41.8 | 7.00E-42 | Verified | *fbpC* | iron | III |
| MW-142AGL001059 | 44.7 | 9.80E-132 | Predicted | *fbp54* | Fibronectin-binding protein / Fibrinogen-binding protein | Fibronectin-binding proteins |
| MW-142AGL001074 | 49.2 | 2.00E-39 | Predicted | *orfM* | putative deoxyribonucleotide triphosphate pyrophosphatase | LOS |
| MW-142AGL001084 | 57.8 | 7.20E-135 | Predicted | *tig/ropA* | Trigger factor, putative | Trigger factor |
| MW-142AGL001085 | 73.2 | 9.80E-157 | Predicted | *tuf* | translation elongation factor Tu | EF-Tu |
| MW-142AGL001127 | 42 | 3.50E-25 | Verified | *CBU_1594* | Coxiella Dot/Icm type IVB secretion system translocated effector | T4SS effectors |
| MW-142AGL001237 | 40.7 | 1.10E-38 | Predicted | *FN3523_1292* | Ribulose-phosphate 3-epimerase | Capsule |
| MW-142AGL001240 | 41.2 | 4.70E-22 | Predicted | *stp* | Serine/threonine phosphatase stp | Serine-threonine phosphatase |
| MW-142AGL001254 | 45.5 | 5.10E-53 | Verified | *CBU_1566* | Coxiella Dot/Icm type IVB secretion system translocated effector | T4SS effectors |
| MW-142AGL001282 | 40.8 | 8.10E-34 | Predicted | *hitC* | ABC transporter related | Haemophilus iron transport locus |
| MW-142AGL001320 | 51.3 | 2.30E-43 | Predicted | *slrA* | peptidyl-prolyl cis-trans isomerase, cyclophilin-type | Streptococcal lipoprotein rotamase A |
| MW-142AGL001326 | 50.9 | 2.00E-69 | Predicted | *hlyD* | probable hemolysin | Hemolysin |
| MW-142AGL001350 | 68.1 | 2.00E-206 | Predicted | *groEL* | chaperonin GroEL | GroEL |
| MW-142AGL001356 | 44.7 | 1.50E-43 | Predicted | *hlyIII* | Hemolysin III | Hemolysin III |
| MW-142AGL001384 | 41.2 | 2.20E-45 | Predicted | *pchA* | salicylate biosynthesis isochorismate synthase | Pyochelin |
| MW-142AGL001395 | 42 | 9.30E-93 | Predicted | *glnA1* | glutamine synthetase | Glutamine synthesis |
| MW-142AGL001409 | 47.7 | 1.40E-56 | Predicted | *KPN2242_15480* | hypothetical protein | LPS rfb locus |
| MW-142AGL001421 | 40.9 | 3.90E-118 | Predicted | *secA2* | protein translocase subunit secA | Accessory secretion factor |
| MW-142AGL001429 | 59.5 | 3.10E-228 | Verified | *clpE* | ATP-dependent protease | ClpE |
| MW-142AGL001439 | 62.1 | 1.90E-66 | Verified | *clpP* | ATP-dependent Clp protease proteolytic subunit | ClpP |
| MW-142AGL001501 | 56.5 | 1.70E-101 | Verified | *efaA* | endocarditis specific antigen | EfaA |
| MW-142AGL001503 | 43.8 | 5.70E-52 | Predicted | *sitB* | Iron transport protein, ATP-binding component | Ferrous iron transport |
| MW-142AGL001517 | 47.1 | 4.90E-270 | Predicted | *FN3523_0021* | carbamoyl-phosphate synthase large chain | Pyrimidine biosynthesis |
| MW-142AGL001518 | 42.2 | 2.60E-77 | Predicted | *Fphi_0805* | carbamoyl phosphate synthase small subunit | Pyrimidine biosynthesis |
| MW-142AGL001521 | 40.1 | 5.50E-55 | Predicted | *kpsF* | arabinose-5-phosphate isomerase | Capsule biosynthesis and transport |
| MW-142AGL001522 | 51.2 | 9.50E-67 | Verified | *kdsA* | 2-dehydro-3-deoxyphosphooctonate aldolase | LPS |
| MW-142AGL001568 | 61 | 1.30E-112 | Predicted | *galE* | UDP-glucose 4-epimerase | Polysaccharide capsule |
| MW-142AGL001595 | 55.5 | 7.40E-261 | Verified | *clpC* | endopeptidase Clp ATP-binding chain C | ClpC |
| MW-142AGL001617 | 46.9 | 9.10E-82 | Predicted | *manA* | mannose-6-phosphate isomerase | Polysaccharide capsule |
| MW-142AGL001619 | 41 | 1.50E-26 | Verified | *flmH* | short chain dehydrogenase/reductase family oxidoreductase | Polar flagella |
| MW-142AGL001629 | 47.3 | 3.40E-227 | Predicted | *lap* | aldehyde-alcohol dehydrogenase protein | Listeria adhesion protein |
| MW-142AGL001630 | 47.1 | 1.80E-150 | Predicted | *ctpV* | cation-transporting ATPase V | Copper exporter |
| MW-142AGL001669 | 40.4 | 8.00E-40 | Predicted | *regX3* | Sensory transduction protein RegX3 | RegX3 |
| MW-142AGL001767 | 49.8 | 1.60E-62 | Predicted | *KPN2242_15480* | hypothetical protein | LPS rfb locus |
| MW-142AGL001771 | 42.9 | 4.00E-47 | Predicted | *mprA* | two component transcriptional regulator, winged helix family | MprA/B |
| MW-142AGL001803 | 40.3 | 3.30E-35 | Predicted | *ddrA* | DrrA | PDIM |
| MW-142AGL001814 | 40.2 | 7.70E-15 | Predicted | *epsH* | exopolysaccharide biosynthesis protein, acetyltransferase | Capsule |
